# Risk assessment model used to predict discharge care after total hip and total knee arthroplasty: A population-based study

**DOI:** 10.1016/j.jor.2024.10.031

**Published:** 2024-10-23

**Authors:** Henrique Alves, Sebastien Di Tommaso, Julien Wegrzyn, Cedric Mabire

**Affiliations:** a Institute of Higher Education and Research in Healthcare - IUFRS, Lausanne University Hospital, University of Lausanne, Lausanne, Switzerland; b University Hospitals of Geneva, Geneva, Switzerland; c Department of Orthopaedic, University Hospital of Lausanne, University of Lausanne, Lausanne, Switzerland

**Keywords:** Total hip arthroplasty, Total knee arthroplasty patient demographics, Predictive models, Discharge destination, Readmission to post-acute care

## Abstract

**Background:**

Transfer to a post-acute care facility or hospital readmission after total joint arthroplasty represent additional costs and increased surgical and health care resource utilization. Accurate prediction of post-acute care factors could help providers to plan the patient's discharge destination and have a positive impact on postoperative outcomes and readmission rates.

**Objective:**

To develop a risk assessment model to predict discharge care after total hip arthroplasty (THA) and total knee arthroplasty (TKA).

**Design:**

A retrospective longitudinal observational study.

**Settings:**

and participants: This study included 209 patients who underwent primary unilateral THA or TKA at a major academic medical center in Switzerland from January 2018 to December 2019.

**Methods:**

A collection of computerized- and paper-recorded data identified the discharge destination, socio-demographic factors, comorbidities, and other factors related to the patient. Univariate and multivariate analyses were performed to describe the predictors of post-surgical discharge destinations.

**Results:**

The characteristics associated with post-acute care after primary unilateral THA or TKA were the absence of a caregiver, advanced age, female gender, presence of walking aids, high ASA score, and comorbidity severity. A prediction model demonstrated that these six characteristics were associated 52 % with discharge to a post-acute care destination.

**Conclusions:**

This study allowed us to identify predictors of discharge to a post-surgical destination. Predictive models can be efficiently used to better predict which patients are predisposed to post-acute care after hospital discharge. Further studies are needed to determine the optimal criteria for different destinations.

**What is already known**.

• The number of joint replacements increases each year, explained by the aging of the population and the demand from young patients for primary arthroplasty. Therefore, surgical and health care resource utilization is also increasing.
• No previous studies have been reported on this topic in French-speaking Switzerland.

**What this paper adds**.

• This study demonstrated that the absence of a caregiver is a key predictor of post-acute care after post-surgical discharge.
• Study recommendations have been established. For THA or TKA, the main criteria for referral to post-acute care were as follows:
  o Demographics: advanced age and female gender
  o Psychosocial and environmental: absence of a caregiver at home and presence of auxiliary aids and service
  o Biophysical: ASA score ≥3 and severity of comorbidities

## 1 Introduction

In Switzerland, 40 % of hospitalized patients underwent surgery in 2018.^1^ Musculoskeletal surgeries are among the most common surgical interventions, with over 220,000 procedures annually in Switzerland.^1^ Orthopedic surgeries often address pathological conditions like osteoarthritis, a chronic and debilitating disease causing adult disability.^2^ Arthroplasty is the primary treatment for osteoarthritis, particularly for hip and knee joints.^3^ This procedure aims to restore independence in daily activities by reducing pain and enhancing physical function.^4^, ^5^, ^6^, ^7^

Following total joint arthroplasty, rehabilitation care is provided to over 75 % of patients.^8^ Rehabilitation aims to alleviate pain, reduce post-surgery complications,^3^ and enhance muscle strength and functionality, thus promoting independence, minimizing disability-related issues, and easing the transition to home life.^9^ This care can be administered in various settings, including rehabilitation centers, post-acute care facilities, or through home healthcare services.^3^^,^^8^^,^^10^^,^^11^

Switzerland witnesses an annual increase in prosthetic procedures, leading to heightened demand for surgical and healthcare resources.^12^, ^13^, ^14^, ^15^, ^16^ This upward trend stems from several factors: an aging population, extended life expectancy, and escalating functional demands in both daily and recreational activities.^7^^,^^17^ The growing number of younger patients seeking primary arthroplasty further contributes to this trend.^12^

These developments necessitate exploration of cost reduction strategies and methods to enhance patient care quality.^17^^,^^18^ Effective approaches include predicting discharge destinations^19^ and establishing pre-defined post-acute care pathways, which can significantly reduce care costs.^20^ Another cost-saving strategy involves identifying post-acute care factors that positively influence postoperative outcomes^19^ and decrease readmission rates.^21^

Early assessment of post-acute care needs serves a dual purpose: it helps identify patients requiring post-acute services^22^ and enables timely interventions to prevent readmissions or unnecessary use of emergency facilities.^23^^,^^24^ This proactive approach not only optimizes resource allocation but also contributes to improved patient outcomes and overall healthcare efficiency.

Conceptualizing patients' bio-psycho-social characteristics related to post-acute care needs can be challenging.^25^ Nurses often overestimate patient capabilities and home environment suitability.^26^ Their discharge assessment skills influence patient care quality.^25^ An evidence-based assessment tool could assist nurses in identifying patients for post-acute care.^22^

The majority of research in this field centers on patient insurance as a successful key predictor of post-acute care needs.^11^^,^^19^^,^^27^, ^28^, ^29^, ^30^, ^31^, ^32^, ^33^ However, the effectiveness of this predictor may vary in different healthcare systems, such as Switzerland's universal healthcare model. In such contexts, other factors may play a more significant role. 10.13039/100014337Furthermore, a notable gap exists in the exploration of socio-cultural factors, such as the availability of caregivers or the absence of home support systems, which could be particularly relevant in diverse healthcare landscapes.

Gaining deeper insights into these socio-cultural elements holds significant potential for improving patient outcomes. A more comprehensive understanding of these factors could enable healthcare providers to better identify and manage pre-surgery risk factors. This proactive approach may, in turn, mitigate the likelihood of post-hospital adverse events.^10^

The limited focus on socio-cultural aspects in existing research underscores the need for more comprehensive studies. Future investigations that encompass both insurance-related and socio-cultural factors could provide a more holistic view of post-acute care predictors. Such research could inform the development of more effective pre-operative risk assessment tools and post-operative care strategies, ultimately leading to improved patient outcomes and more efficient healthcare resource allocation.

The aim of this study was to identify the predictive factors for postoperative care after an acute in-hospital stay during the pre-surgical consultation in order to anticipate the patient's discharge care after total hip arthroplasty (THA) or total knee arthroplasty (TKA).

## 2 Methods

### 2.1 Design

The design of the study was retrospective and longitudinal.

### 2.2 Patient selection and consent process

This retrospective study utilized data from patients who had previously signed the general consent form for research participation. The process for obtaining general consent is defined and approved by the clinical research committee of the institution. As part of this process, potential participants receive the consent form by mail, which they can review, sign, and return via mail if they agree to participate.

For this specific retrospective study, a careful procedure was followed. Eligible patients were first identified based on the inclusion criteria. Subsequently, verification was conducted to ensure that each selected patient had a signed general consent form on file. Patients without a signed consent were excluded from the study to ensure ethical compliance.

This approach aligns with the institution's guidelines for retrospective studies and ensures that all data used in the research was obtained with proper consent.

### 2.3 Data collection

A dedicated data analyst extracted structured hospitalization data spanning from January 1, 2018, to December 31, 2019. Simultaneously, the lead researcher (HA) manually examined electronic patient records to gather unstructured information. When faced with uncertainties or ambiguities, consultation was sought from the head of hip and knee reconstruction in the Orthopaedic Surgery Department (JW). The research employed a longitudinal approach, collecting data from two key points: Time 0, marking the initial pre-operative consultation, and Time 1, indicating the patient's discharge location. This design allowed for comprehensive tracking of patient journeys from pre-surgical assessment through to post-operative care destinations.

### 2.4 Endpoints

The study focused on two main outcomes. The primary outcome examined the post-acute care discharge pathway, distinguishing between patients returning home without rehabilitation and those receiving rehabilitation assistance. Secondary outcomes encompassed various biophysical and psychosocial factors, as detailed in Table 1. These factors provided a comprehensive view of the patient's condition and circumstances, offering insights into the factors influencing post-acute care needs following surgery.

**Table 1:** Biophysical and psychosocial factors.

Variable	Variable type	Data measurement
Age	Sociodemographic	Age of the patient in years before the intervention
Gender	Sociodemographic	Patient gender, male or female
Height	Health data	Size in cm
Weight	Health data	Weight in kg
BMI	Health data	BMI in kg/m^2^
ASA score	Health data	American Society of Anesthesiologists score, level between 1 and 4
Psychiatric history	Health data	Classified into subcategories according to the International Classification of Diseases, 10th revision (ICD-10)
Medical background	Health data	Classified in ICD-10 subcategory
Surgical history	Health data	Classified in ICD-10 subcategory
Ongoing treatment	Health data	Classified in subcategories according to the classification of the Anatomical Therapeutic Chemistry
Type of intervention envisaged	Health data	Between THA and TKA
Planned destination upon exit	Health data	Between return home, treatment and rehabilitation centre, or home help
Native country	Sociodemographic	Ranked by permanent foreign resident population
marital status	Sociodemographic	Between single, married, widowed, divorced, and separated
Assurance level	Sociodemographic	Between basic, semi-private, or private insurance
Professional activity	Sociodemographic	Classification according to the Federal Statistical Office
Caregiver	Sociodemographic	Caregiver present/absent
Home help	Sociodemographic	Home help present/absent
Type of residence	Sociodemographic	Between an apartment, a house, or a medico-social establishment
Native language	Sociodemographic	Classified according to the same classification as the country of origin
Auxiliary means	Health data	Use or not of an auxiliary means when travelling
Actual/planned length of stay	Health data	Duration in days
Level of training completed	Sociodemographic	Classified according to the register of diplomas of the national framework

BMI = body mass index; ASA = American Society of Anesthesiologists.

### 2.5 Statistical analysis

The study employed standard methods for descriptive analyses. Multivariate logistic regressions were utilized to develop predictive models based on sociodemographic and clinical characteristics observed during pre-surgical consultations. The process of identifying potential predictors of post-acute care involved a sequential variable selection approach.

Initially, correlations between variables were evaluated using biserial correlation coefficients. This was followed by simple linear regression analysis between explanatory variables (potential predictive factors) and the explained variable (post-acute care).

The selection of predictors for each model involved applying backward elimination criteria. The discriminatory power of each logistic regression model was assessed using the area under the curve.

The relationship between predictive factors and discharge destination was explored through both simple and multivariable logistic regressions. Results were presented using regression coefficients, accompanied by 95 % confidence intervals and p-values. All statistical analyses were conducted using Stata® software, version 16.1.

This approach allowed for a comprehensive examination of factors influencing post-acute care needs, providing insights into the complex interplay of patient characteristics and care outcomes. The rigorous statistical methodology ensured the reliability and validity of the findings, contributing to a deeper understanding of post-surgical care pathways.

### 2.6 Ethical considerations

The study was approved by our institutional review board (CER-VD -2020-02525).

## 3 Results

The study period from January 1, 2018, to December 31, 2019, identified 446 patients who underwent THA or TKA at the institution's total joint registry. Exclusion criteria removed 237 patients: 155 due to THA or TKA related to trauma, infections, or oncology diseases, and 82 for revision arthroplasty procedures. The final study cohort comprised 209 patients, with 114 (54 %) receiving THA and 95 (46 %) receiving TKA. Based on discharge destinations, the cohort was divided into two groups: 70 patients (33 %) returned home without rehabilitation care, while 139 patients (67 %) required rehabilitation assistance either at home or in a facility.

The patient cohort had a mean age of 68 ± 10 years (SD = 10), consisting of 116 women (56 %) and 93 men (44 %). Demographics showed 132 Swiss patients (63 %), 115 married individuals (55 %), and 145 unemployed persons (69 %), of whom 129 (89 %) were retired. Further characteristics included 188 patients with basic insurance (90 %), 155 living in apartments (74 %), 150 moving without auxiliary means (72 %), 192 without home assistance (92 %), and 115 with a caregiver (55 %). Post-arthroplasty, 75 patients (36 %) required post-acute care, with 52 (25 %) transferred to rehabilitation facilities.

Clinical characteristics revealed a mean BMI of 27 kg/m2, with 131 patients (63 %) having an ASA score of 2. The median number of comorbidities was 6, with all patients presenting “diseases of the osteo-articular system, muscles or connective tissue”, specifically osteoarthritis, as per the study's inclusion criteria. Additionally, 67 % had “diseases of the circulatory system”, with 58 % receiving “cardiovascular system” drug treatments.

Table 2 presents the associations between sociodemographic and clinical characteristics of patients receiving post-acute care. The analysis compared patients returning home without rehabilitation against those requiring post-acute care at home or in rehabilitation facilities. Correlation testing for each variable preceded the regression model analyses.

**Table 2:** Univariate correlation: variables associated with post-acute care.

Characteristic	Without post-acute care	With post-acute care	Biserial correlation
	*n* = 139	*n* = 70	
***Age, mean in years (SD)***	65 (8.97)	74 (9.04)	0.45
***Age categorized***			0.43
40–60 years, n (%)	42 (30.2)	4 (5.7)	
60–80 years, n (%)	92 (66.2)	43 (61.4)	
80–100 years, n (%)	5 (3.6)	23 (32.9)	
***Female gender***	65 (46.8)	51 (72.9)	0.25
***Being single, n (%)***	11 (10.4)	8 (28.6)	0.21
***No caregiver, n (%)***	39 (28.7)	53 (77.9)	0.47
***Presence of home help, n (%)***	0	17 (25)	0.43
***With auxiliary means, n (%)***	16 (14.7)	32 (49.2)	0.37
***Without professional activity, n (%)***	78 (56.1)	67 (95.7)	0.4
***Median ASA score (min–max)***	2 (1–4)	2 (1–4)	0.28
***ASA score ≥ 3, n (%)***	29 (20.9)	32 (45.7)	0.26
***Median of comorbidities (min–max)***	6 (1–14)	9 (2–16)	0.4
***Comorbidities present according to ICD-10***			
ICD6 Diseases of the nervous system, n (%)	28 (20.1)	34 (48.6)	0.31
ICD7 Diseases of the eye, n (%)	7 (5)	15 (21.4)	0.24
ICD9 Diseases of the circulatory system, n (%)	84 (60.4)	56 (80)	0.34
ICD14 Diseases of the genitourinary system, n (%)	28 (20.1)	26 (37.1)	0.15
ICD17 Birth defects, n (%)	0	4 (5.7)	0.2
***Median ATC treatments (min–max)***	3 (0–11)	4 (0–15)	0.2
***Usual treatments according to ATC***			
Digestive system and metabolism, n (%)	51 (36.7)	43 (61.4)	0.23
Blood/blood-forming organs, n (%)	28 (20.1)	36 (51.4)	0.32
Cardiovascular system, n (%)	66 (47.5)	55 (78.6)	0.3

ASA = American Society of Anesthesiologists; ICD-10 = International Classification of Diseases, 10th revision; ATC = Anatomical Therapeutic Chemical classification.

**Fig. 1:**
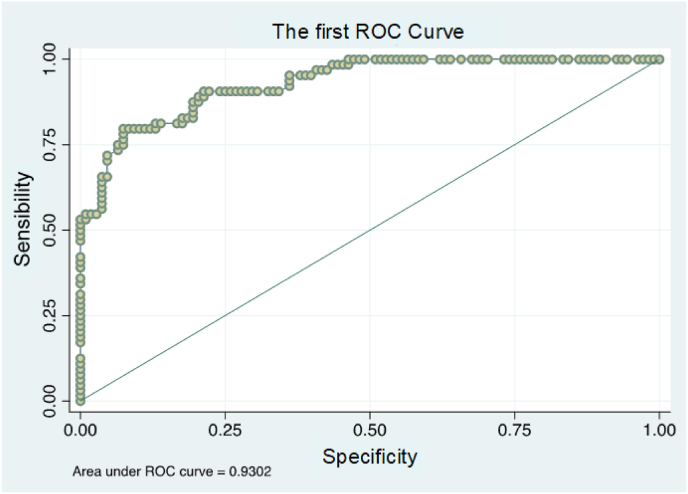
Area under the curve: first prediction model. ROC = receiver operating characteristic.

**Fig. 2:**
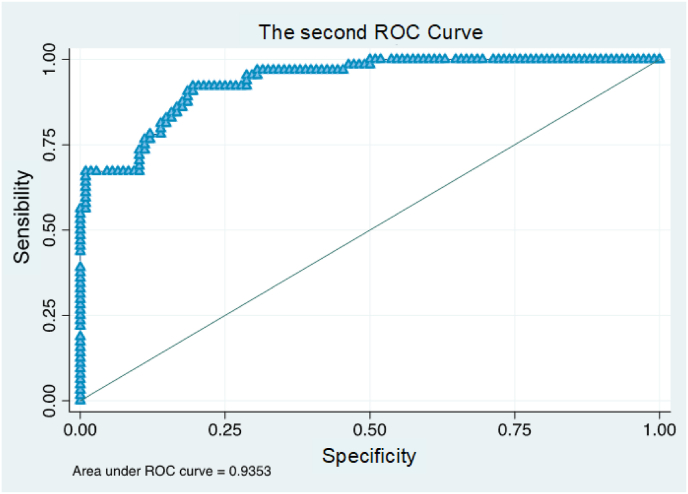
Area under the curve: second prediction model. ROC = receiver operating characteristic.

Two prediction models were defined, one having ASA variables and the other comorbidity variables. The first model explained more than 51 % of the association with post-surgical discharge destinations (Table 3). The second model explained more than 52 % of the association (Table 4). The area under the curve for the first model was 0.93 (Fig. 1) and for the second model was 0.94 (Fig. 2).

The study developed two distinct prediction models. One model incorporated ASA variables, while the other utilized comorbidity variables. The first model demonstrated a strong association with post-surgical discharge destinations, explaining over 51 % of the relationship, as detailed in Table 3. The second model showed a slightly higher explanatory power, accounting for more than 52 % of the association, as presented in Table 4. Both models exhibited robust predictive capabilities. The area under the curve for the ASA-based model reached 0.93, as illustrated in Fig. 1. The comorbidity-based model achieved a marginally higher value of 0.94, depicted in Fig. 2. These results indicate the high discriminatory power of both models in predicting post-surgical discharge destinations.

**Table 3:** First logistic regression model.

Adj. R-squared^a^ = 0.51	Univariate analysis		Multivariate analysis	
N of obs = 172 R-squared = 0.5196	*Coefficient* [95 % CI]	*p-*value	*Coefficient* [95 % CI]	*p-*value
Home help present	0 (omitted)			
Lack of caregiver	2.17 [1.5; 2.9]	0.01	3.07 [1.9; 4.2]	0.01
Presence of auxiliary means	1.73 [1; 2.4]	0.01	1.54 [0.4; 2.7]	0.01
Female gender	1.12 [0.5; 1.7]	0.01	1.43 [0.4; 2.5]	0.01
ASA score	1.08 [0.5; 1.6]	0.01	1.38 [0.5; 2.2]	0.01
Age	0.12 [0.1; 0.2]	0.01	0.14 [0.1; 0.2]	0.01

ASA = American Society of Anaesthesiologists; CI = confidence interval.

a Adjusted R^2^ manually calculated.

**Table 4:** Second logistic regression model. ].

Adj. R-squared^a^ = 0.52	Univariate analysis		Multivariate analysis	
N of obs = 171 R-squared = 0.53	*Coefficient* [95 % CI]	*p-*value	*Coefficient* [95 % CI]	*p-*value
Lack of caregiver	2.17 [1.5; 2.9]	0.01	2.97 [1.8; 4.1]	0.01
Presence of auxiliary means	1.73 [1; 2.4]	0.01	1.51 [0.4; 2.6]	0.01
Female gender	1.12 [0.5; 1.7]	0.01	1.29 [0.3; 2.3]	0.01
Comorbidity	0.3 [0.2; 0.4]	0.01	0.32 [0.1; 0.5]	0.01
Age	0.12 [0.1; 0.2]	0.01	0.13 [0.1; 0.2]	0.01

CI = confidence interval.

a Adjusted R^2^ manually calculated.

## 4 Discussion

The objective of this study was to identify patient characteristics associated with post-acute care after a THA or TKA. Therefore, the most important finding of this study was the predictive factors, which were, in order of importance, advanced age, female gender, absence of a caregiver, the presence of walking aids, a high ASA score, and severity of comorbidities.

Numerous studies have consistently identified advanced age and female gender as key predictors of post-acute care needs.^3^^,^^11^^,^^27^^,^^30^^,^^32^, ^33^, ^34^, ^35^, ^36^, ^37^, ^38^, ^39^, ^40^, ^41^, ^42^, ^43^, ^44^ The influence of advanced age on post-surgical discharge destinations may be attributed to its association with increased vulnerability and functional decline. Regarding gender, women are more frequently transferred to rehabilitation facilities.^7^^,^^27^^,^^28^^,^^33^^,^^35^^,^^37^, ^38^, ^39^, ^40^^,^^42^^,^^44^

The influence of gender on post-acute care needs warrants deeper investigation. Social dynamics may play a role, as societal expectations and familial responsibilities often differ between men and women. Furthermore, the connection between osteoporosis and functional decline merits consideration, albeit with the acknowledgment that sarcopenia affects both genders.

The presence of a home caregiver significantly enhances post-orthopedic surgery rehabilitation. Multiple studies^35^^,^^41^^,^^45^ highlight this psychosocial factor as a strong predictor of post-acute care needs, a finding corroborated by the multivariate analysis presented in Table 1, Table 2 These insights could shape healthcare interventions by emphasizing pre-surgical evaluation and addressing of caregiver availability. Patients without daily assistance might benefit from increased community support or home care services, potentially leading to improved rehabilitation outcomes and reduced postoperative care costs. Research by Sattler et al.^43^ indicates that patients lacking home assistance post-surgery face a sixfold increase in the likelihood of requiring post-acute care.

The absence of a caregiver correlates with two additional predictors of post-acute care needs: living alone^19^^,^^27^^,^^28^^,^^36^ and insufficient family support.^36^^,^^38^ These factors may signify feelings of loneliness, which, while difficult to quantify, can significantly influence post-acute care requirements. The complex interplay of these social factors underscores the need for further prospective research focusing on living arrangements and social support networks.

Preoperative functional capacity is a key predictor of post-acute care needs.^3^^,^^38^^,^^39^ Patients with reduced mobility may require more intensive inpatient treatment,^3^ while the use of walking aids before surgery can indicate functional decline and predict rehabilitation needs.^35^

The ASA score stands out as a widely acknowledged predictor of post-acute care needs, as evidenced by multiple studies.^19^^,^^27^^,^^31^^,^^37^^,^^39^^,^^40^ Its validity stems from its ability to correlate preoperative conditions with postoperative outcomes.^45^ Research indicates that THA or TKA patients with an ASA score of 3 face a 3.5 times higher likelihood of requiring long-term care post-discharge compared to those scoring 1. This probability escalates dramatically, increasing 11-fold for patients with an ASA score of 4.^27^ Consequently, an ASA score of 3 or above serves as a strong indicator of potential post-acute care requirements.^27^^,^^31^^,^^37^^,^^40^

The impact of specific comorbidities on post-acute care needs shows variation across different studies.^30^^,^^33^^,^^35^^,^^42^ However, a consistent trend emerges: patients with multiple comorbidities demonstrate a higher likelihood of discharge to rehabilitation facilities. The multivariate analysis in this study corroborates the number of comorbidities as a significant predictor of post-acute care needs. Following THA or TKA procedures, the probability of requiring post-surgical care increases in proportion to the number of comorbidities present.^32^

Interestingly, certain predictors of post-acute care identified in previous research did not show significance in the current study. Body Mass Index (BMI), for instance, which several studies found significant,^28^^,^^30^^,^^36^^,^^40^^,^^41^ did not emerge as a significant factor in this analysis. This finding aligns with the results of Rudasill et al..^31^ The discrepancy might be explained by the potential unreliability of BMI measurements in older adults experiencing sarcopenia, a condition characterized by loss of muscle mass.^46^

5. The assessment of extended rehabilitation likelihood following total joint arthroplasty frequently involves prediction tools. Notable examples include the PLAN tool (Predicting Location after Arthroplasty Nomogram) utilized in Barsoum et al., 's 2020 study and the ARISE tool (Arthroplasty Rehabilitation Initial Screening Evaluation) employed in Sattler et al., 's 2020 research. Of particular relevance is the RAPT (Risk Assessment and Predictor Tool), developed by Oldmeadow et al., in 2003, which produced results aligning closely with the current study.
6. Oldmeadow et al.'s analysis of data from 530 THA or TKA patients in an Australian tertiary care hospital identified seven variables significantly linked to post-surgical discharge destination. These variables encompassed age, gender, preoperative walking distance, walking assistance, home help, caregiver presence, and the patient's preferred destination. Notably, four of these variables - age, gender, walking assistance, and caregivers - correspond with the findings of the present study.
7. The RAPT tool demonstrated high accuracy in predicting discharge destinations, ranging from 75 % to 88 %.^47^, ^48^, ^49^ Its user-friendly nature and consistent performance across multiple countries have been validated in subsequent research,^50^ further underscoring its reliability and applicability in diverse healthcare settings.

## 5 Limitations

Our study had several constraints. A primary limitation was data availability. Patient data for this study was collected exclusively from existing hospital records, utilizing information obtained after patients had provided their general consent for research purposes. The study's methodology was designed to analyze only the information contained within these records, without any additional contact with patients for data collection. This approach, while limiting in some aspects, ensured consistency in data collection and minimized potential recall bias. Another absent social factor, crucial for predicting post-acute care needs, was the patient's preferred post-surgical discharge destination. Additionally, the absence of a validation cohort prevented us from verifying our predictive model on a separate group of patients, limiting its generalizability.

## 6 Conclusions

The study reveals the significance of preoperative patient-reported characteristics in indicating post-surgical discharge destinations for THA and TKA procedures. Caregiver availability emerges as a crucial factor in determining post-acute care needs. This finding is further supported by several other influential predictors, including advanced age, female gender, reliance on assistive devices, elevated ASA scores, and the presence of multiple comorbidities.

Improving patient outcomes necessitates a comprehensive preoperative evaluation process that actively engages the patient. Such an approach allows for a more thorough assessment of individual needs and potential post-surgical challenges.

The research underscores the need for future studies to develop a more refined predictive model. This model should incorporate additional variables identified in existing literature, while also being adaptable to various post-surgical discharge destinations. By doing so, healthcare providers can better anticipate and prepare for individual patient needs, potentially leading to improved post-operative care and recovery outcomes.

This enhanced predictive capability could contribute significantly to healthcare resource allocation, patient care planning, and ultimately, to better post-surgical experiences for patients undergoing THA and TKA procedures.

## Legal framework

This study was conducted in accordance with Article 34 of the Swiss Federal Act on Research involving Human Beings (Human Research Act, HRA).

## Nature of the study

This research consists of extraction and analysis of pre-existing data from medical records. No new data was directly collected from patients for this study.

## Justification for absence of individual consent

In accordance with Article 34 of the HRA, individual patient consent for this specific study was not solicited for the following reasons:

1. Obtaining consent would have been impossible or disproportionately difficult.
2. The research project involves minimal risk.
3. The interest of science outweighs the interest of individuals in deciding on the use of their personal data.

## Data protection

All data used in this study were treated confidentially and anonymized. No information that could identify patients has been or will be disclosed in the study results or in any resulting publications.

## CHUV general consent

It is important to note that all participants included in this study had previously signed the general consent form of the Lausanne University Hospital (CHUV). This general consent allows for the use of patients' health-related personal data and biological material for research purposes, in compliance with applicable laws and ethical standards.

## CRediT authorship contribution statement

**Henrique Alves:** Conceptualization, Methodology, Data curation, Formal analysis, Writing – original draft, Writing – review & editing. **Sebastien Di Tommaso:** Conceptualization, Methodology, Data curation, Writing – review & editing. **Julien Wegrzyn:** Conceptualization, Methodology, Writing – review & editing. **Cedric Mabire:** Conceptualization, Methodology, Formal analysis, Writing – review & editing, All authors have read and approved the final version of the manuscript submitted for publication.

## Conflict of interest

JW received royalties from Dedienne santé; is a paid consultant for Stryker, Lima Corporate, Mathys, and DePuy Synthes; is on the editorial board of the *Journal of Arthroplasty*; and is an academic editor for *Swiss Medical Weekly*.

## Ethical Statement

Hereby, Professor Cedric Mabire consciously assure that for the manuscript risk assessment model used to predict discharge care after total hip and total knee arthroplasty: A population-based study the following is fulfilled:

1) This material is the authors' own original work, which has not been previously published elsewhere.
2) The paper is not currently being considered for publication elsewhere.
3) The paper reflects the authors' own research and analysis in a truthful and complete manner.
4) The paper properly credits the meaningful contributions of co-authors and co-researchers.
5) The results are appropriately placed in the context of prior and existing research.
6) All sources used are properly disclosed (correct citation). Literally copying of text must be indicated as such by using quotation marks and giving proper reference.
7) All authors have been personally and actively involved in substantial work leading to the paper, and will take public responsibility for its content.

The violation of the Ethical Statement rules may result in severe consequences.

I agree with the above statements and declare that this submission follows the policies of Switzerland as outlined in the Guide for Authors and in the Ethical Statement.

## Declaration of relevant financial Disclosure(s)

☒ The authors declare that they have no relevant financial relationships with ineligible companies or personal relationships that could have appeared to influence the work reported in this paper.

☐The authors declare the following relevant financial relationships with ineligible company(ies) within the past 24 MONTHS and/or personal relationships which may be considered as potential competing interests: ∗*Please also indicate if the relationship has ended or if the relationship still exists.*

## Ethical approval

This study was approved by [insert name of the competent ethics committee], which confirmed that the study complies with the requirements of Article 34 of the HRA and the ethical principles of medical research.

## Additional information

The use of data in this study is strictly limited to the purposes outlined in the research protocol. The researchers are committed to maintaining the highest standards of data protection and respect for patient privacy throughout the research process.

## Funding sources

This research received no specific grant from any funding agency in the public, commercial, or not-for-profit sectors.
